# Multi-Modal Nano Particle Labeling of Neurons

**DOI:** 10.3389/fnins.2019.00012

**Published:** 2019-02-01

**Authors:** Lilac Amirav, Shai Berlin, Shunit Olszakier, Sandip K. Pahari, Itamar Kahn

**Affiliations:** ^1^Schulich Faculty of Chemistry, Technion – Israel Institute of Technology, Haifa, Israel; ^2^Department of Neuroscience, Ruth and Bruce Rappaport Faculty of Medicine, Technion – Israel Institute of Technology, Haifa, Israel

**Keywords:** MRI, iron oxide nanoparticles, light microscopy, contrast agents, magneto fluorescence nanoparticle labeling

## Abstract

The development of imaging methodologies for single cell measurements over extended timescales of up to weeks, in the intact animal, will depend on signal strength, stability, validity and specificity of labeling. Whereas light-microscopy can achieve these with genetically-encoded probes or dyes, this modality does not allow mesoscale imaging of entire intact tissues. Non-invasive imaging techniques, such as magnetic resonance imaging (MRI), outperform light microscopy in field of view and depth of imaging, but do not offer cellular resolution and specificity, suffer from low signal-to-noise ratio and, in some instances, low temporal resolution. In addition, the origins of the signals measured by MRI are either indirect to the process of interest or hard to validate. It is therefore highly warranted to find means to enhance MRI signals to allow increases in resolution and cellular-specificity. To this end, cell-selective bi-functional magneto-fluorescent contrast agents can provide an elegant solution. Fluorescence provides means for identification of labeled cells and particles location after MRI acquisition, and it can be used to facilitate the design of cell-selective labeling of defined targets. Here we briefly review recent available designs of magneto-fluorescent markers and elaborate on key differences between them with respect to durability and relevant cellular highlighting approaches. We further focus on the potential of intracellular labeling and basic functional sensing MRI, with assays that enable imaging cells at microscopic and mesoscopic scales. Finally, we illustrate the qualities and limitations of the available imaging markers and discuss prospects for *in vivo* neural imaging and large-scale brain mapping.

## Introduction

A central goal in neuroscience research is the development of imaging methodologies for longitudinal single neuron interrogation in the intact animal. High-resolution light-microscopy (LM) imaging of neurons *in vivo* enables structural and functional mapping, as well as remote optical control with exquisite spatiotemporal resolution; shedding light on some of the most fundamental questions related to neural morphology and function in health and disease. However, LM offers but a glimpse of the brain *in vivo*, typically providing access to very small fields-of-view and depths of the mammalian brain (Silva, 2017; Zong et al., 2017). Thus, non-invasive imaging modalities that can reveal the structure and function of neurons in the entire brain, i.e., at the mesoscale, could provide the complementary means needed for this ambitious endeavor. Magnetic resonance imaging (MRI) is particularly attractive to meet this goal, owing to its soft-tissue imaging capabilities, and the ability to image the entire organ non-invasively and repeatedly over a long period of time, through the intact skull and at any depth, with no known adverse impact on the tissue.

As commonly used, MRI provides an indirect measure of the brain’s structure and function, owing to the manner by which signals are acquired — by perturbing the alignment of the highly abundant hydrogen atoms nuclear spins found in this tissue. In structural MRI, this signal is indistinguishable between the intra- versus extra-cellular environments and across cell types. Similarly, the contrast mechanism commonly measured by functional magnetic resonance imaging (fMRI) is also indirect (Logothetis, 2008; Kim and Ogawa, 2012). Intrinsic signals for fMRI arise from the ratio between oxygenated and deoxygenated hemoglobin, a mesoscopic metabolic measure, rather than measures of electrical activity or intracellular physiological events, such as Ca^2+^ concentration changes or neurotransmitter release. In recent years, several technological advances, including magnetic fields significantly higher than those commonly used in clinical settings, novel radio-frequency coils with a large number of densely-spaced small coil elements, and the use of unique dielectric materials, allow to indirectly track the activity of smaller and smaller populations of neurons in the brain (Ugurbil, 2016). Despite these innovations, the signals are still minute, of relatively poor signal-to-noise ratio (SNR) and indirect. To overcome these limitations, means to increase contrast of defined targets are under development, in particular MRI-compatible contrast-agents that are designed to detect specific molecular targets in the brain (Mukherjee et al., 2017; Ghosh et al., 2018). As contrast-agents are used routinely in clinical MRI scans, novel classes of contrast agents suitable for use in humans will certainly enhance and expand the capabilities of the technique. We propose that bi-functional materials that serve as hybrid contrast-agents for multiple imaging modalities at once, notably LM and MRI, will open new fronts for structural and functional whole-brain imaging at higher resolution and with target specificity.

The design and synthesis of bi-functional materials is an active research area with a significant impact on a wide range of technological applications (Corr et al., 2008; Gao et al., 2009; Suh et al., 2009; Qin and Bischof, 2012; Cheng et al., 2014). From an imaging point-of-view, combining several different properties onto a single agent can functionalize it towards many types of imaging and detection modalities; greatly extending its diagnostic, and potentially therapeutic, value. Indeed, emerging multifunctional contrast agents have been shown to label cancerous cells (Sailor and Park, 2012; Dawidczyk et al., 2014; Sanna et al., 2014) or genetically-modified cells *in vivo* (Kim et al., 2008; Muthu et al., 2014; Ortgies et al., 2016) for detection by MRI and validation by LM. Target selective imaging is a prerequisite for image-guided interventions (including surgery and ablation therapy) (Li, 2014). Beyond imaging, multifunctional contrast agents may also include pharmacological agents to concomitantly visualize and treat diseases in an *all-in-one* therapeutic and diagnostic (theranostic) approach (Yoo et al., 2011; Lim et al., 2015), for which there is a growing number of examples (Gao, 2018). Specifically, iron oxide nanoparticles are utilized for magneto-responsive therapy, where the responsiveness of the nanoparticles to an external magnetic field is used in order to increase the accumulation of the particles in a target tissue (magnetic targeting), or for exogenous physical stimuli release of cargo gene or drug molecules (Lee et al., 2015). However, this field is still in its infancy, with significant limitations in *bona fide* labeling of defined cellular targets and at obtaining sufficient particle accumulation at desired locations for gaining higher resolutions; notably to the single-cell level.

Cell-targeted contrast agents could provide the means to increase target-specificity and resolution by adhering and accumulating around or within cells. Extracellular contrast agents are typically aimed at reversibly binding proteins exposed to the extracellular-milieu. However, owing to protein-turn-over, limited expression of the target-protein, expression of some proteins in a large variety of cell types and limited membrane surface-area (Saka et al., 2014), such agents do not typically provide sufficient contrast of defined-cells; especially not for prolonged durations (Mukherjee et al., 2017). Intracellular contrast agents, on the other hand, may bypass several of these limitations and, thereby, potentially provide an extended imaging time-window. For instance, membrane expression levels and protein turn-over are less likely to impact the latter. In addition, the intracellular space (i.e., cytoplasm) greatly exceeds that of the membrane surface, allowing for the accumulation of larger amounts of contrast agents and, consequently, to provide higher signals. Intracellular accumulation also slows washout of the agent, thus extending the imaging periods, and it may also localize the activity of a therapeutic agent, or enable a weak drug (e.g., with a high median effective dose) to become efficient exclusively in the desired cellular population where the drug has been concentrated.

To meet their full potential, intracellular contrast-agents and their further development should benefit from better understanding of their cellular uptake mechanisms. Of particular interest is information on the modes of cellular uptake, their efficiency and kinetics, subcellular distribution of contrast agents following uptake, saturation concentrations, clearance and, notably, toxicity. Gaining control over these parameters will open the door towards novel basic and clinical applications.

A key requirement towards meeting this goal is the ability to track and validate the intracellular accumulation of contrast agents with high spatiotemporal resolution, significantly higher than what is currently afforded by MRI, and over an extended period of time. Here, multifunctional contrast agents that can be detected by both LM and MRI are particularly useful, lending themselves to achieve this task. Fluorescence imaging provides exquisite high-resolution means to explore and validate the different features of cellular uptake. It can be used to assess the accumulation of specific contrast agents at the cellular, subcellular, protein or even single molecule level, and at very high temporal resolution. To this end, several magneto-fluorescent hybrid systems are currently under development.

## Magneto Fluorescence Hybrid Nanoparticles

In order to combine LM and MRI, appropriate agents for both imaging modalities first need to be considered based on their signal strength, toxicity, stability and size. Several classes of MRI-compatible contrast agents are available (Geraldes and Laurent, 2009); with iron oxide nanoparticles meeting most of these requirements, namely provide a strong and stable MRI-signature, with little effects on cellular physiology (e.g., Uchiyama et al., 2015; and see review Shen et al., 2017). Consequently, iron oxide nanoparticles are commonly employed in the field. In particular, these have been rendered bi-modal by their conjugation to organic fluorescent dyes, such as fluorescein isothiocyanate, rhodamine B, and Cy5.5; all commonly employed bright markers for LM (Wysocki and Lavis, 2011). However, organic dyes may undergo rapid photobleaching and/or photochemical degradation (shortening the imaging time-window), or produce cytotoxic byproduct (e.g., reactive oxygen species; Zheng et al., 2014) and damage to the biological system under investigation. To address the instability of organic dyes, several reports describe encapsulation of dyes within silica to provide protection (Ow et al., 2005; Piao et al., 2008), but this provides only a moderate protection and comes at the cost of larger particles size. Quantum dots (QDs), inorganic fluorescent semiconductor nanoparticles, are an attractive alternative (Kim et al., 2004; Medintz et al., 2005). QDs have a high molar extinction coefficient and fluorescence quantum yield, broad absorption, and narrow tunable emission spectra, in addition to excellent temporal stability and resistance to photobleaching. These make QDs particularly advantageous over other fluorescent agents. However, coupling of QDs to MR contrast agents, specifically iron oxide nanoparticles, is not straightforward, and likely the reason why this has not been commonly achieved. This is mainly because a direct contact between the semiconductor and magnetic domain can lead to strong electronic coupling and strong attenuation of the QDs’ fluorescence. Indeed, traditional heterodimer structures, namely placing the semiconductor QD directly on the surface of an iron oxide nanoparticle, are not sufficiently bright for optical imaging (Selvan et al., 2007). Fluorescence quenching can be minimized, or prevented entirely, if effective separation is achieved (Boldt et al., 2011;Bigall et al., 2012; Feld et al., 2015; Harris et al., 2016). Hence, alternative synthetic strategies for the fabrication of magneto-fluorescent materials include conjugation of separate nano-constructs, or co-encapsulation into organic structures or inorganic materials (Kim et al., 2006; Kim and Taton, 2007; Insin et al., 2008; Park et al., 2008; Roullier et al., 2008; Erogbogbo et al., 2010; Fan et al., 2010; Kas et al., 2010; Di Corato et al., 2011; Shibu et al., 2013; Cho et al., 2014; Lee et al., 2015). These conceptual designs are expected to prevent undesirable interactions within the hybrid that could abrogate the respective properties.

**FIGURE 1:**
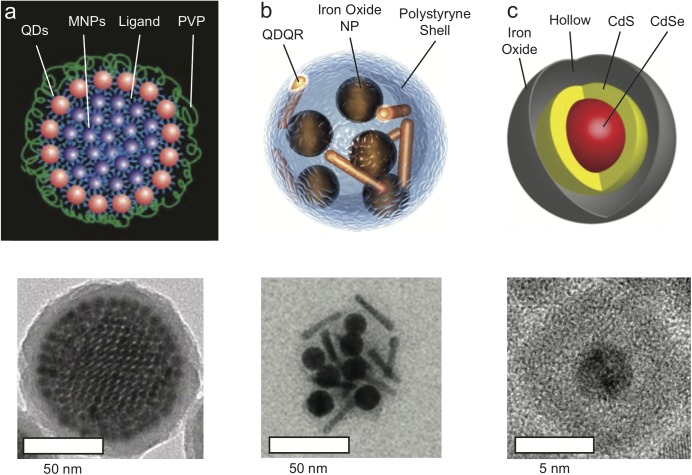
Magneto-Fluorescent Nanoparticles. **(a)** Schematic illustration (top) and TEM micrograph (bottom) of co-assembled super-nanoparticles consisting of closed-packed Fe_3_O_4_ magnetic nanoparticles “core” surrounded by a “shell” of fluorescent CdSe@CdS quantum dots (QDs). Following coating with silica, the superstructure obtain an average diameter of 100 nm. Adapted with permission from Chen et al. (2014). Copyright (2014) Springer Nature. **(b)** Schematic illustration and TEM micrograph of magneto-fluorescent nanohybrid produced by two-step co-encapsulation of iron oxide nanoparticles and fluorescent CdSe@CdS quantum dots/quantum rods (QDQRs) into polystyrene shell, with a 50 nm diameter structures. Adapted with permission from Feld et al. (2015). Copyright (2015) John Wiley and Sons. **(c)** Schematic illustration and TEM micrograph of magneto-fluorescent yolk-shell hybrid structures composed of CdSe@CdS core that is encapsulated within a hollow-Fe_2_O_3_ shell, with a 10 nm diameter. Adapted with permission from Pahari et al. (2018). Copyright (2018) American Chemical Society.

Two prominent examples of such designs are presented in Figure 1a,b. Bawendi and co-workers (Chen et al., 2014) developed colloidal superstructures comprised of close-packed magnetic nanoparticle cores that are fully surrounded by a shell of fluorescent QDs, and the core-shell superparticle is coated with a protective silica shell. These super-nanoparticles exhibit high magnetic content (with T_2_ relaxivity of 402.7 mM^-1^S^-1^) along unperturbed fluorophore loading, with an overall diameter of ∼100 nm. Weller and co-workers (Feld et al., 2015) used polystyrene to co-encapsulate iron oxide nanoparticles with quantum rods, thereby preserving the fluorescence and magnetism of the separate components, with particle diameters ranging from 74 to 150 nm, and relaxivity of 164 mM^-1^S^-1^.

**FIGURE 2:**
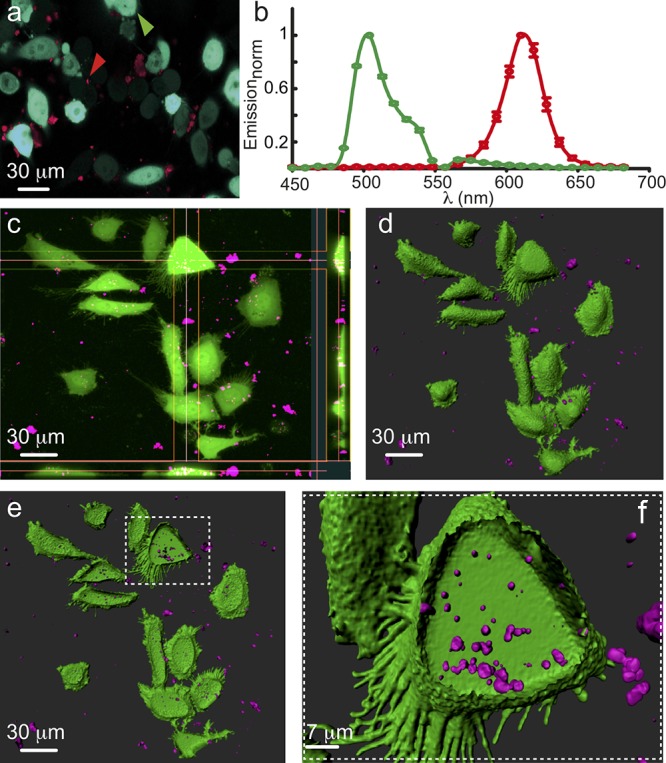
Intracellular Accumulation of Nanoparticles. **(a)** Spectral micrograph showing HeLa cells transfected with cytosolic GFP (cyan filled cells, green arrowhead) incubated with nanoparticles (NPs; red dots and arrowhead). **(b)** Emission spectra of GFP (green plot) and NPs (red plot). **(c)** Projection micrograph (from Z-stack) and orthosteric views showing NPs (magenta) located within individual cells. **(d**,**e)** Three dimensional rendering (Imaris) of **(c)** depicting outer surface (green) of the cells along with the inner volume of the cells containing NPs (magenta). **(f)** Higher power image showing a single cell filled with at least few dozens of NPs.

Recently, we presented a novel design strategy for the fabrication of ultra-small (∼15 nm hydrodynamic size) magneto-fluorescent nanoparticles (Pahari et al., 2018). *In lieu* of conjugation or co-encapsulation of the separate magneto and fluorescent components into an insulating matrix, both were combined into a single entity with a unique morphology. The optically active semiconductor QD was encapsulated directly into a hollow paramagnetic iron oxide shell that serves as the MRI contrast agent (Figure 1c). Despite their small size, these nanoparticles provide contrast enhancement with a relaxivity of 304 mM^-1^S^-1^, making them comparable to the much bigger aforementioned systems, and superior to commercial contrast agents such as Fe_3_O_4_ nanoparticles (26.8 mM^-1^S^-1^). This so-called “yolk-shell” morphology prevents the undesired interactions within the hybrid that would diminish the properties of the semiconductor and magnetic domains. Furthermore, this architecture offers a high level of tunability with respect to size, composition and surface functionalization. The photoluminescence quantum yield of the yolk-shell nanoparticles was typically ∼16% (at 450 nm excitation light), comparable to the ∼12% photoluminescence quantum yield (at 405 nm) of the silica coated core-shell superparticles. This yield was sufficient for single particle tracking (Chen et al., 2014), and for optical microscopy imaging of nanoparticle clusters within single cells, or of 3D cell cultures (as seen in Figure 2; Pahari et al., 2018). The decrease in photoluminescence quantum yield compared with that of free QDs, that could be as high as 94%, is attributed to the overlapping absorption with that of the iron oxide.

Noteworthy is the fact that for the morphologies presented in Figure 1, the fluorescent QDs may be altered with no effect on overall size, surface characteristics and magnetic properties of the particle. Hence, we anticipate that future hybrids will replace the Cd-based semiconductor with non-toxic and biocompatible alternatives and incorporate materials with efficient near-infrared (NIR) optical activity in both absorption and fluorescence; wavelengths more suitable for *in vivo* applications. This adjustment will also minimize the spectral overlap between the absorption of the core and that of the hollow shell, and is expected to improve the photoluminescence quantum yield.

An interesting alternative for multimodal imaging is provided by incorporation of manganese ions into the QD, hence offering the semiconductor itself paramagnetic characteristics (Sitbon et al., 2014). These structures may serve as T1-contrast agents, influencing the longitudinal relaxation time, whereas superparamagnetic iron oxide nanoparticles are employed as T2-contrast agents, influencing the transverse relaxation time.

## Intracellular Accumulation of Nano-Particles

Particle-size is a critical criterion that will determine the mechanism by which it will be internalized into the cell, with major influences on cellular uptake efficiency and kinetics, and subcellular distribution (He et al., 2010; Huang et al., 2010; Verma and Stellacci, 2010; Shang et al., 2014; Zhang et al., 2015). Note that this refers to the overall size of the structure, including the inorganic and organic shells. The modes of internalization can vary quite extensively, ranging from processes such as opsonization and phagocytosis (Gustafson et al., 2015), through clathrin/caveolar-mediated endocytosis (Hachani et al., 2017) and receptor-mediated endocytosis (Qiao et al., 2012), to macro- and pinocytosis. The size of a contrast agent will also impact the means by which it escapes from the endosomal/lysosomal compartments and enters the cytoplasm (for a review see, Oh and Park, 2014).

A significant factor for *in vivo* imaging is the effect of the hydrodynamic diameter of the contrast agent on its resident circulation time (Kievit and Zhang, 2011). Nanoparticles with a hydrodynamic diameter of less than 10 nm, i.e., smaller than the pore size of the glomerulus, are subjected to rapid excretion and thus have relatively short dwell-time in the blood stream (Chapman et al., 1999; Olmsted et al., 2001; Choi et al., 2007; Liu et al., 2013; Ehlerding et al., 2016). On the other hand, nanoparticles with an overall size larger than 100 nm may be recognized and removed quickly from the blood stream by the reticuloendothelial system (opsonization by white blood cells, namely macrophages), which will also result in short circulation time (Reimer and Tombach, 1998). Ultrasmall superparamagnetic iron oxide nanoparticles that are smaller than 50 nm can escape phagocytosis to some extent with a prolonged circulation time (Shen et al., 1993; Wang et al., 2001; Li et al., 2005). An overall size of 15 nm was found to result with relatively longer circulation time, affording prolonged MRI signal enhancement effects for over an hour, and collection of high-resolution MR images of blood vessels (Kim et al., 2011). Therefore, longitudinal measurements in the intact animal that depend upon longer circulation time will require unique adjustment and optimization of the overall size of the agent. Together, nanoparticles should not be too small, to prevent rapid central nervous system clearance, and should not be too large, to avoid uptake by macrophages. Thus, the synthetic strategy of choice is critical for appropriate functionality of the magneto-fluorescent hybrid structure. In this regard, the yolk-shell strategy may present a convenient design for experiments that require smaller dimensions.

In addition to size, the shape, surface chemistry and aggregation or agglomeration habits of the nanoparticles will also influence the type of internalization process (Albanese and Chan, 2011). In particular, surface coating is found to play an important role in successful delivery of nanoparticles into the intracellular environment (e.g., see Figure 2), and it may additionally offer opportunities for selective labeling of relevant targets. The surface of our yolk-shell magneto-fluorescent nanoparticles is that of the iron oxide shell, rather than silica or polystyrene. This allows implementation of the vast accumulated knowledge that is available in the literature for surface coating and functionalization of iron oxide for the purpose of biological compatibility. Hence, insights on cell internalization and accumulation of the yolk shell magneto fluorescent nanoparticles, which are acquired via utilization of the fluorescent marker, could be directly implemented for similar iron oxide therapeutic nanoparticles. A prominent example relies on distinctive biochemical characteristics of target diseases such as tumor or inflammatory tissue, with surface-functionalization that is designed for improved cell and tissue specific distribution of nanoparticles for localized therapeutic effect, and minimal whole-body toxicity (Lee et al., 2015). In addition, along side functionality of the iron oxide nanoparticles as therapeutic hyperthermic agents (Petryk et al., 2013; Hervault and Thanh, 2014), or for controlled drug release through the application of an external magnetic field (Yoo et al., 2011), the QD core could easily be replaced with Au (Shevchenko et al., 2008; Jain et al., 2012) or FePt (Gao et al., 2008) for cancer therapy. Note that such functionalities are hindered or lost entirely if the iron oxide nanoparticles are coated within thick silica or polystyrene shell.

In the course of our work, the CdSe@CdS@hollow-Fe_2_O_3_ yolk-shell magneto-fluorescent nanoparticles (MFNP) were functionalized first with Tiron (disodium 4,5-dihydroxy-1,3-benzenedisulfonate) for their incorporation to cells (Korpany et al., 2013). This resulted with stable and well suspended water-dispersed nanoparticles of 14–16 nm hydrodynamic particle size (as determined by dynamic laser scattering analysis). Alternative ligand coatings such as polymer or PEG resulted with drastic increase of the hydrodynamic size, with no added stability. Yet, the intracellular accumulation of Tiron-coated nanoparticles was found to be variable and, at times, limited. Hence, dopamine was examined as an alternative coating (Sherwood et al., 2017). We, and others, envisioned the dopamine-coat to play several critical roles. First, it was expected to increase the solubility of the nanoparticle. Second, it could be recognized by membrane receptors or transporters to promote cell-specific receptor-mediated endocytosis and internalization of the particles into cells of interest, neurons in particular. Lastly, this coat was also envisioned to play a role in reducing the ability of extracellular proteins to adhere to the nanoparticles, thereby negating their increase in size and clearance by engulfing cells. Though successful to some extent, we have obtained weak LM signals from cells suggesting only moderate cytoplasmic fill. To further enhance endocytosis, we turned to established chemical reagents that are commonly employed for the introduction of foreign material into cells such as DNA. The MFNP were incubated with the transfection reagent lipofectamine, glycerol; a water soluble and hygroscopic lipid, calcium phosphate precipitates and spermidine; a naturally occurring polyamine commonly used in biolistic transfection of neurons. Strikingly, and despite the common features of the reagents, preliminary observations showed that spermidine yielded the best results; with cells displaying detectable intracellular particles with clear evidence of aggregation (∼1 μm in diameter; Figure 2). Notably, external modifications and coatings did not affect the functionality of the encapsulated QDs, as evident by their emission fluorescence spectrum outside or within cells (Figure 2a,b).

The observation of aggregates was surprising, as the nanoparticles consistently remain monomeric in solution *in vitro*. This therefore suggested to us that the particles are undergoing agglomeration, despite the dopamine coat, and this process is likely mediated by the physiological and/or intracellular conditions. Though unsettling at first, we quickly realized that this process could be beneficial for intracellular trapping of the nanoparticles because as small particles may easily enter the cell, so can they exit it. Deliberate and controlled aggregation may serve for entrapping and accumulating nanometer particles within the cell more readily, in turn providing stronger MRI and fluorescent signals, not to mention increasing the stability of the particles over time due to lower exposure to degradation. This observation also suggests that with further design, nanoparticles could be engineered to undergo activity-dependent aggregation. A relevant and interesting example to explore is aggregation that is induced by the binding of a certain biological moiety that surges in an activity-dependent manner, such as Ca^2+^-ions (Okada et al., 2018). In this regard, we envision that the coat of the nanoparticles could include small molecules or protein fragments (e.g., Li et al., 1999; Atanasijevic and Jasanoff, 2007; Henig et al., 2011) that bind Ca^2+^, e.g., calcium-specific aminopolycarboxylic acid such as 1,2-bis(2-aminophenoxy)ethane-N,N,N’,N’-tetraacetic acid (BAPTA) or Calmodulin, respectively. A plausible scenario would be that following neural activity and sharp rise in cytoplasmic Ca^2+^-concentration, intracellular nanoparticles would bind Ca^2+^, thus altering the reactivity of the coating. Ca^2+^ binding may influence the effective columbic repulsion of the coating, or enhance the affinity and binding to a secondary moiety on the surface of surrounding nanoparticles. Regardless the exact mechanism, it is expected to change the particles’ dispersivity in a manner that would result with strong aggregation. This aggregation, once it exceeds a certain size, is expected to result in a detectable MR signal localized to the cell. As Ca^2+^-binding is reversible, this phenomenon would also be reversible, providing MRI-readout for neuronal activity.

## Conclusion

Intracellular magneto-fluorescent contrast agents are of great significance for the development of imaging methodologies for single cell longitudinal measurements in the intact brain. These bi-functional markers enable coupling between high resolution LM with the non-invasive mesoscale imaging capabilities of MRI. Yet the roadmap towards enhancement of MRI signals from select specific cells requires improved understanding of cellular internalization and accumulation mechanisms. In particular, modes of cellular uptake, their efficiency and kinetics, subcellular distribution of contrast agents following uptake, clearance and, notably, toxicity need to be better understood and accurately characterized. These are all influenced, and at times dictated, by the size, shape and surface chemistry of the contrast agent nanoparticles, and should be properly considered when choosing the synthetic strategy. We envision that insights on the correlation between the agents’ characteristics and their intracellular distribution would enable manipulation of the latter for improved specificity, and ultimately for functional readout of neuronal activity.

## Ethics Statement

All of the experiments involving animals were conducted in accordance with the United States Public Health Service’s Policy on Humane Care and Use of Laboratory Animals and study protocol was approved by the Institutional Animal Care and Use Committee of Technion – Israel Institute of Technology.

## Author Contributions

LA, SB, and IK developed the concepts described in this work and wrote the manuscript. SO, SP, and SB carried out the experiments. All authors analyzed the results described in the manuscript and approved the final version of the manuscript. This work is in partial fulfilment for the Degree of Doctor of Philosophy (Ph.D.) for SO.

## Conflict of Interest Statement

The authors declare that the research was conducted in the absence of any commercial or financial relationships that could be construed as a potential conflict of interest.
